# The challenge of monitoring elusive large carnivores: An accurate and cost-effective tool to identify and sex pumas (*Puma concolor*) from footprints

**DOI:** 10.1371/journal.pone.0172065

**Published:** 2017-03-08

**Authors:** Sky Alibhai, Zoe Jewell, Jonah Evans

**Affiliations:** 1 Nicholas School of the Environment, Duke University, Durham, North Carolina, United States of America; 2 JMP Division, SAS, Cary, North Carolina, United States of America; 3 Department of Mechanical and Aerospace Engineering, North Carolina State University, Raleigh, North Carolina, United States of America; 4 Texas Parks and Wildlife, Austin, Texas, United States of America; U.S. Geological Survey, UNITED STATES

## Abstract

Acquiring reliable data on large felid populations is crucial for effective conservation and management. However, large felids, typically solitary, elusive and nocturnal, are difficult to survey. Tagging and following individuals with VHF or GPS technology is the standard approach, but costs are high and these methodologies can compromise animal welfare. Such limitations can restrict the use of these techniques at population or landscape levels. In this paper we describe a robust technique to identify and sex individual pumas from footprints. We used a standardized image collection protocol to collect a reference database of 535 footprints from 35 captive pumas over 10 facilities; 19 females (300 footprints) and 16 males (235 footprints), ranging in age from 1–20 yrs. Images were processed in JMP data visualization software, generating one hundred and twenty three measurements from each footprint. Data were analyzed using a customized model based on a pairwise trail comparison using robust cross-validated discriminant analysis with a Ward’s clustering method. Classification accuracy was consistently > 90% for individuals, and for the correct classification of footprints within trails, and > 99% for sex classification. The technique has the potential to greatly augment the methods available for studying puma and other elusive felids, and is amenable to both citizen-science and opportunistic/local community data collection efforts, particularly as the data collection protocol is inexpensive and intuitive.

## 1 Introduction

### 1.1 Terminology

Trail: An unbroken series of footprints made by one animal

Footprint: A single impression made by a foot

Track: Commonly used in the literature to describe both an individual footprint and a trail.

### 1.2 The challenge and necessity of studying puma populations

The puma (*Puma concolor*; also commonly known as mountain lion, cougar, panther, catamount) is the most widely distributed free-ranging land mammal in the Americas, ranging from Northern Canada to the Southern Andes. Like other cryptic carnivores with large territories, puma populations are notoriously difficult to study [1]. Despite the challenges, agencies responsible for managing pumas are often tasked with estimating their populations [2]. The need for reliable data on puma populations has led to the development of a range of research approaches including: capture-recapture [3,4], extrapolation of hunter harvest data [2], comparison of harvest and demographic data [5], camera trap surveys [6,7], aerial snow trail counts [8], and several variations of track surveys [9–11]. However, there are shortcomings with each of these methods that prevent wide-scale adoption.

While estimates of absolute abundance (the total number of animals in a population) are generally preferred over indices of relative abundance, the former usually require the identification of individual animals. A common method for identifying individual pumas in a free-ranging population, capture-mark-recapture, is prohibitively expensive for wide-scale practical use [12]. Consequently, researchers often use indices of relative abundance (footprint or trail counts, hunter harvest, etc.) which, while more affordable can be less reliable, and rarely define the true relationship between the index and the actual population [4].

### 1.3 The risks and penalties of invasive monitoring techniques

Invasive survey methods such as capturing pumas with dogs or snares are often used when estimates of absolute population size are needed, but can result in direct physical injury or death [13,14].

There are also less obvious consequences of invasive survey methods. For example, the repeated stimulation of the mammalian adrenocortical axis by external stressors in standard capture-mark-release procedures can result in profoundly negative effects on a range of physiological systems, including the immune and reproductive systems [15–17]. The process of immobilization, and particularly repeated immobilization, can also have unexpected effects on behaviour, including reduced ranges in black bears [18], reduced body condition in Polar bears [19], and changes in sex ratios of offspring in water voles [20]. In addition, radio and GPS collars can cause injuries or even death in various species, including: African wild dog [21], kit foxes, [22], mule deer [23] and black rhino [24]. A thorough review is provided by Murray & Fuller [25].

Conservationists are also increasingly aware of welfare and ethical issues in monitoring [26] and how invasive approaches might also compromise the validity of data they gather.

### 1.4 The use of indirect signs for monitoring

Indirect signs (footprints, scat, nests, etc.) can be the most effective and least expensive way to detect many animals [12,27]. Animal footprints are much more frequently encountered in the field than the animals themselves, and have served as the basis for population indices and estimators [12,28,29]. Footprint surveys (also called track surveys) are also non-invasive; the animal need not be seen, captured, or handled.

### 1.5 Non-invasive approaches used to study puma populations

Researchers have used at least three non-invasive methods to study puma populations: camera traps, genetic analysis of hair and scat, and footprint surveys [30]. Camera traps have been used to identify individual animals by analysis of spots and stripes [31,32], but may underestimate carnivore numbers [7]. Because pumas lack distinguishing marks, accurate identification by camera-trap images with large sample sizes has proven difficult [6,33]. Genetic identification of individuals from scats and hair has also been used [34–36], but puma scats can be difficult to find in the field. Some researchers have successfully used scat detection dogs to improve detection rates [37] however, scat dogs require considerable training and care, factors that may be prohibitive for some managers.

### 1.6 Previous attempts to classify footprints by individual and sex

There are many accounts in the literature of efforts to solve this challenge by identifying individuals of a wide range of species from their footprints, including: fisher, *Martes pennanti* [38]; tiger, *Panthera tigris* [39–42]; black rhino, *Diceros bicornis* [43]; white rhino, *Ceratotherium simum* [28]; snow leopard, *Panthera uncia* [41]; jaguar, *Panthera onca* [44]; brown bear *Ursus arctos* [45,46]; and European pine marten, *Martes martes* [47]. Additionally, the extraordinary ability of indigenous experts to identify individuals from footprints was reported by Stander et al. [48].

Footprints have also been used to classify tiger and puma by sex. Early work focused on the shape description of footprints [40,49,50]. This was superseded by a more quantitative approach based on simple comparison of measurements [51–54]. More recent work has focused on the statistical analysis of one or several measurements [42,55,56].

### 1.7 Previous puma footprint research

Researchers have attempted to use footprints to identify and sex individual pumas [57–60]. Smallwood and Fitzhugh [60] were the first, to our knowledge, to publish an objective mathematical method for discriminating individual puma footprints using measurements. Their method was based on measurements taken from footprint tracings made in the field and was successfully tested with nine free-ranging pumas. Grigione et al. [57] refined the technique developed by Smallwood and Fitzhugh [60] and successfully tested it with a known population of 10 pumas. Lewison et al. [58] validated the methodology presented by Smallwood and Fitzhugh with footprints made from plaster casts taken from the feet of 13 pumas. However, the rigidity of plaster casts differs from the flexible feet of living animals, and may not be an ideal substitute for natural field conditions. While these projects showed initial success, their small sample sizes and complex methodologies continue to limit broad scale field application.

### 1.8 The Footprint Identification Technique (FIT)

The Footprint Identification Technique (FIT) software enables the identification of pumas by sex and individual using a classification algorithm based on measurements of distance, angle and area taken between anatomically derived points on the footprint. The software was developed for black rhino monitoring [28,43] but has subsequently been adapted for a wide range of species [61].

To the best of our knowledge, the FIT presented here is the first system for identifying individual puma that is based on a large data training-set (535 footprints from 35 unique animals), and the first to have all analytical processes encapsulated in a software package with an integrated graphical user interface.

The FIT puma algorithm has built upon previous puma footprint classification approaches in the following respects:

#### 1.8.1 Used a large training-set to develop a best-fit algorithm

The FIT software has derived a best-fit algorithm from a large training-set of footprints, in this case from 35 known individual puma (known individual puma used in previous studies: Smallwood and Fitzhugh [60] n = 9, Grigione [57] n = 3, Lewison [58] n = 13). The FIT algorithm specifies which footprint measurements (variables) are able to discriminate between individuals using a robust cross-validated discriminant analysis that feeds into a Ward’s clustering model.

#### 1.8.2 Extracted more data from each footprint

The FIT extracted more data from each footprint, thereby increasing the potential resolution and accuracy for individual classification. The software generated 123 morphometric variables from each footprint, including areas, lengths, and angles (variables analyzed in previous studies: Smallwood and Fitzhugh [60] n = 11, Grigione [57] n = 9, Lewison [58] n = 17) The large number of variables were used to develop a more robust training dataset than was previously possible.

#### 1.8.3 Analysed more footprints per animal

To build the initial algorithm training-set database, the FIT used a mean of between 14 and 16 left hind footprints from each animal in order to adjust for individual footprint variability.

#### 1.8.4 Developed an integrated software interface

The FIT employs a user-friendly integrated software interface to a new customized statistical model, providing minimal risk of subjective interpretation.

#### 1.8.5 Integrated algorithm validation

The FIT software provides integrated algorithm validation in the form of sequential data holdout testing, by randomly apportioning the data into training and test sets.

#### 1.8.6 Provided a standardized protocol for data collection

Data collection uses a simple, standardized digital protocol for photographing footprints [28].

The overall aim of this research was to demonstrate the potential utility of a new monitoring tool for widespread application in large felid populations, using the puma as a model. This was successfully met.

## 2. Materials and methods

### 2.1 Ethics statement

No invasive techniques were used in this research. Footprint images were collected from captive puma in zoos, animal rescue facilities and animal sanctuaries, all on private land. The owners of the captive puma facilities gave permission to study on their sites. No further formal or informal approvals or permits were needed from an animal ethics committee because all procedures carried out were non-invasive..

### 2.2 Safety statement

When working with captive habituated pumas, two handlers were present at all times, safety gates were employed to ensure that the animals were kept out of areas when footprints were being photographed.

### 2.3 The methodological basis of the Footprint Identification Technique (FIT)

The basis of the individual classification methodology has been described in full by Jewell et al. [62] and Alibhai et al. [28]. Jewell et al. (62) provides an additional video description (S2 Video) of the entire methodology and analytical processes in FIT, using the cheetah (*Acinonyx jubatus*) as an example.

The FIT is able to identify individuals from their footprints on the basis of morphometric variation, in the form of footprint measurements, between them. The initial classification algorithm is extracted from a training set of footprints from known individuals. The algorithm specifies those footprint measurements that are able to discriminate between individuals using a robust cross-validated discriminant analysis that feeds into a Ward’s clustering model [63].

The footprint identification technique is species specific because each species has a unique foot morphology. Thus, each species has a unique algorithm, extracted from a training dataset of footprints from known individuals, in this case puma. Once the species algorithm has been field-validated, it can be applied to identify and monitor unknown free-ranging individuals of that species.

Classification algorithms in FIT are based on Fisher’s canonical variates [64] that generate centroid plots [43]. A forward stepwise regression selects the footprint measurements that have the highest discriminatory power, according to their F-ratios. Stepwise regression is an approach to selecting a sub-set of effects for a regression model, in which a model is generated by progressively adding or removing variables based on the t-statistics of their estimated coefficients.

**Fig 1:**
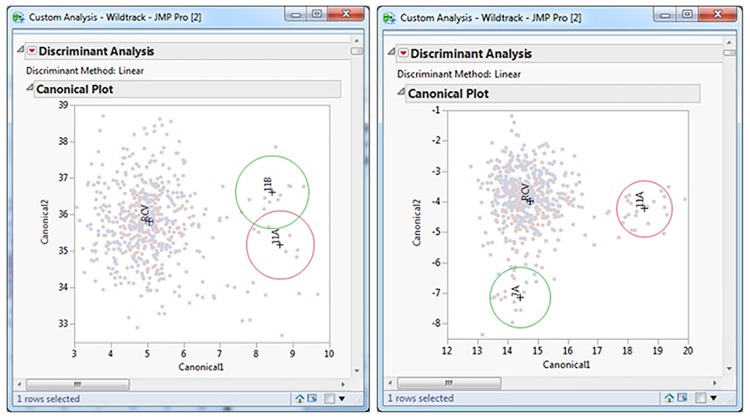
Pairwise comparisons. The figure shows the outcome of a pair-wise comparison of trails from the same individual (A) and two different individuals (B) based on a customized model in JMP software. The classifier incorporated into the model is based on the presence or absence of overlap between the ellipses. Note that the analysis is performed for each pairwise comparison in the presence of a third entity, i.e. the reference centroid value (RCV).

The first two canonical variates are then constructed, from the stepwise selected measurements, to map the trails in a two dimensional space. The centroid values (multivariate least-square means) and 95% confidence interval ellipses are plotted for each trail. This combination provides the classifier for individual identification. If the 95% ellipses overlap, then these two testing trails belong to the same individual. If they don’t overlap, they belong to two individuals (Fig 1). However, we found that the distance between the centroids also depends on the matrix of within-group variations and the relative-position vector of the centroids, so that any changes to a testing set (adding or removing individuals) could change the positions of the centroid values and their ellipses. To eliminate this potential problem and ensure that the distance between centroids was an effective classifier, we [43] proposed two modifications. First, we limited the centroid plot comparison to pairs of trails, comparing only two trails at one time. Second, we developed a "reference centroid value (RCV)" entity, built from all the other known individuals in the library. This functioned as a reference point in the canonical space, to stabilize the location of any test groups with respect to each other [28,62].

We used a standard linear discriminant analysis, coded within the FIT software, to classify by sex.

Three elements of the FIT model must be adjusted to optimize the output algorithm from a training set:

#### 2.3.1 The number of footprint measurements (variables)

Too many footprint measurements may lead to increased separation of trails that are known to be from the same animal (self-trails) and too few footprint measurements may lead to overlap of ellipses of trails known to be from different animals (non-self trails), resulting in an estimate of too few individuals. The puma algorithm was optimized with 14–20 measurements.

#### 2.3.2 The contour probability

This is used to determine the size of the ellipse (the confidence interval around the centroid value), which in turn determines if there will be overlap or not with the ellipse from the other test set in the pairwise comparison.

#### 2.3.3 The threshold value (Ward distance)

Using the above elements, we generated a cluster dendrogram in JMP using the Ward distance. The threshold value that gave the highest level of classification accuracy was used to generate a predicted value for the number of puma in the dataset of known individuals.

The above elements, once optimized, describe the best-fit algorithm for the footprints taken from captive puma, which, after validation using comprehensive sequential holdback trails, can be used to predict the number of puma from unknown populations.

The FIT software runs as an add-in to JMP data visualization software, using the JMP Scripting Language (JSL), that can be customized for any species [65].

### 2.4 Collecting footprint images

The protocol for collection of footprint images of large carnivores is described in manuscript and video in Jewell et al. [62] (Supplementary material S2)

To summarize, the first step in collecting reference images from captive puma is to lay a sand path for them to walk over. Common builder’s sand is acceptable, laid to a depth of about 1cm, and width of 2-3m giving the animal plenty of space to walk comfortably. Captive animals are often most comfortable walking along a perimeter fence, or familiar path. Garden tools are used to rake and flatten the sand, and sprinkle with water if too dry.

There are several critical steps necessary for the success of the protocol. First, sand paths must be prepared correctly and the animal led over the sand at a normal relaxed walking pace. When photographing the footprints, the photographer must be directly overhead of the center of the print. Often it is useful to have a second observer to check this. Lastly, it is essential that the photographer (or an assistant, who might be an expert tracker) be able to identify a left hind puma footprint on the ground, and have the skill to follow the trail of footprints forwards or backwards along the line of travel.

#### 2.4.1 Footprint collection from captive puma

We collected footprints from 35 captive pumas in 10 different captive facilities across the USA including: Capital of Texas Zoo (n = 1), the Center for Animal Research and Education (CARE) (n = 5), Fort Worth Zoo (n = 2), Houston Zoo (n = 2), In-Sync (n = 10), Maine Wildlife Park (n = 1), NW Trek (n = 2), Pride Rock (n = 2), Wild Animal Orphanage (n = 1), and Wildlife Rescue & Rehabilitation (n = 13).

The purpose of collecting footprints from captive animals was to build the reference dataset for the creation of the FIT algorithm. The FIT has been designed to provide optimal accuracy without the need to collect prints from all four feet. Specifically, we collected left hind footprints only. Hind footprints were preferred because they frequently step on top of (register with) front prints and the left side was chosen arbitrarily. We were able to identify left hind footprints by observing clear morphological characteristics in the footprints (i.e., asymmetry in toe position). Puma feet are asymmetrical in the same manner as the human hand, where the middle finger (toe 3) is longer than the rest. The thumb (toe 1) does not make contact with the ground or register in the footprint. The toes also have a tendency to be slightly skewed to the inside of the trail. Hind footprints tend to be longer and narrower than the fronts. The heel pad is usually narrower and smaller. Hind prints are also more symmetrical than the fronts.

**Fig 2:**
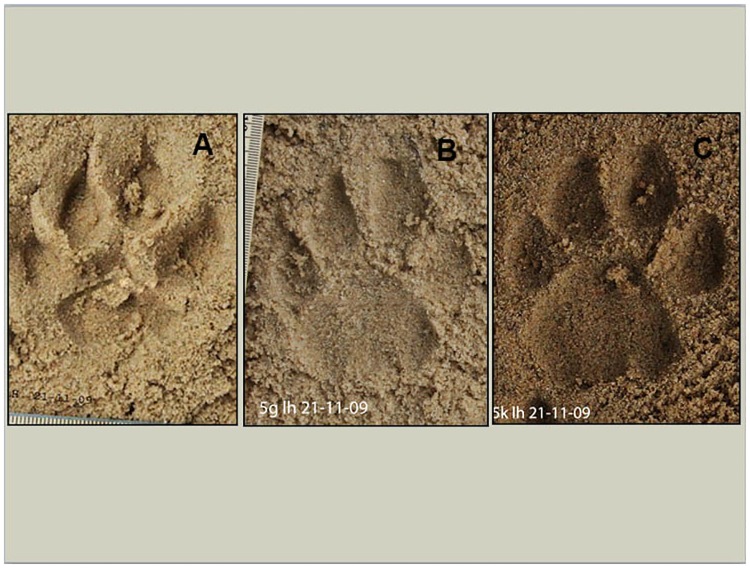
This figure shows three puma footprints of poor (a), acceptable (b), and excellent (c) quality. The main difference is the clarity of the outline of the heel and toes.

Only footprints with clearly visible toe and heel edges were used. Footprints exhibiting distortion were discarded (Fig 2)

**Fig 3:**
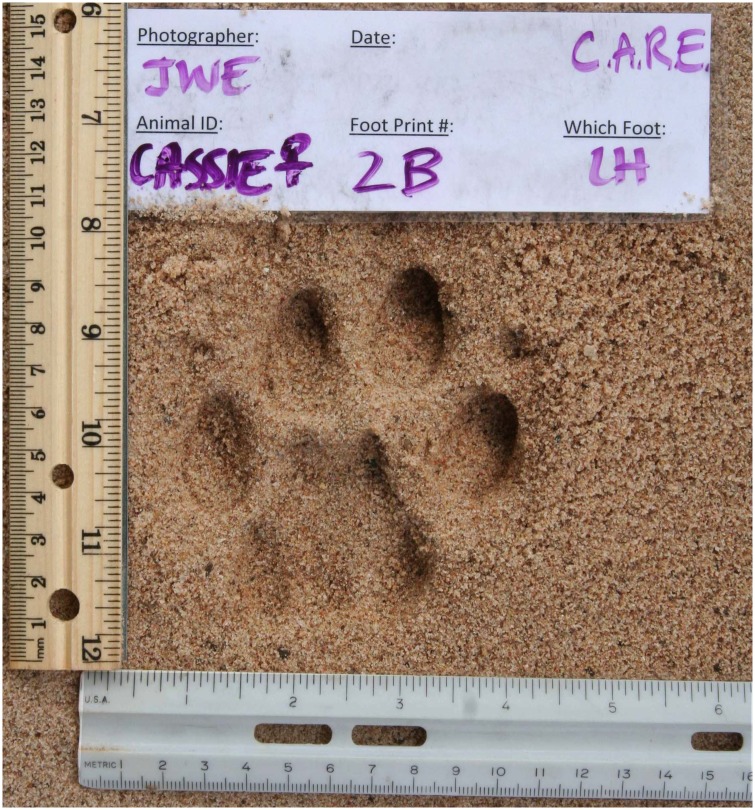
An excellent quality puma footprint, photographed to the FIT protocol. A metric scale is placed to the left and posterior edge of the footprint, and a photo ID slip containing details of the footprint and image is included. The image is taken from directly overhead.

We photographed footprints according to the FIT protocol [62] that requires images be taken from directly above the footprint, a ruler should be placed along the X and Y-axes, and a photo ID slip included in the image (Fig 3) which should include (if known) footprint number, trail number, date, time and location point. Since a scale (ruler) was included in each image, there was no specification for the height at which an image should be taken, only that the image, photo ID slip and scale should fill the camera frame. No level was used, but we recommend inexperienced photographers engage a second person to observe that the camera lens is angled parallel to the ground. Natural lighting conditions varied and so footprints were shaded before photography to avoid partial-shadows or challenges with contrast. Camera pixel resolution and lens settings must be such that mm increments on the ruler and text on the photo ID slip are clearly visible.

#### 2.4.2 Free-ranging pumas

We tested the FIT with footprints from free-ranging pumas to validate the FIT for monitoring unknown free-ranging animals and to confirm that substrates *in situ* would provide sufficient quality of prints.

Following the same photo protocols, footprint images were obtained from free-ranging pumas in Texas and California. To ensure that each trail came from a unique individual, we did not collect more than 1 trail from each geographic region.

### 2.5 Using FIT to classify by individual

#### 2.5.1 Extracting features from footprint images

The following process has been described in detail both in manuscript and video formats for cheetah [62] and will be summarized here.

**Fig 4:**
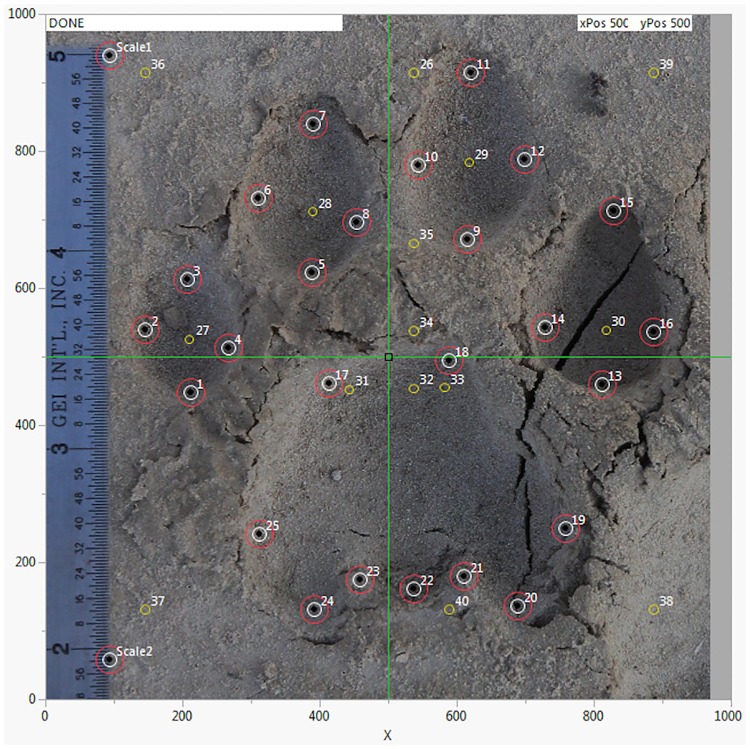
A puma footprint showing the placement of 25 landmark points (red circles) and 15 points derived from them and generated by the FIT script (yellow circles). The landmark points and derived points are numbered in one sequence, providing 40 total points from which measurements (variables) of the footprint are made.

Footprint images are labeled according to the photo ID slip in the image, and filed on the desktop. Statistical analyses are performed in the FIT software, an add-in to JMP data visualization software. The FIT software add-in is opened in JMP and the first footprint image prepared for processing in the ‘Image Feature Extraction’ window. The footprint is rotated and adjusted for depth if necessary and two scale points are identified. Twenty-five landmark points are then placed sequentially on the footprint guided by the feature extraction template on the left of the window. The script then generates a further fifteen ‘derived’ points to augment the number of measurements taken (Fig 4). All data from the Photo ID slip are entered into the image feature extraction window. The x.y coordinates for each landmark and derived point, and one hundred and twenty eight variables including distance, area and angle, are then calculated by the FIT and exported to a JMP datatable.

This process is repeated for each footprint image. All the rows in the database are then copied, and pasted below the database in the same table. This duplication set is called the Reference Centroid Value (RCV) and acts to stabilize the footprint identification technique model for subsequent pairwise comparison of footprint trails. The mathematical explanation of this process is detailed in the appendix of Jewell et al. [43].

#### 2.5.2 Development of the footprint identification technique algorithm for the puma

Each footprint generates a single row of 128 measurements of distances, angles and areas (Table 1). S1 Table provides the full dataset of footprint measurements. From the total data training-set of all the footprints we generate an algorithm, based on those measurements that is able to discriminate between individuals [62].

**Table 1:** The number of variables extracted from the footprint images as lengths (L), angles and areas.

V1 *	L 01–03	V33	L 03–07	V65	L 14–15	V97	L 02–37
V2	L 05–07	V34	L 07–11	V66	L 15–16	V98	L 02–36
V3 *	L 09–11	V35	L 11–15	V67 *	L 13–16	V99	L 26–36
V4	L 13–15	V36	L 15–19	V68	L 17–19	V100	L 11–26
V5	L 02–04	V37	L 03–25	V69	L 18–25	V101 *	L 11–39
V6	L 06–08	V38 *	L 17–27	V70	L 27–28	V102	L 16–39
V7	L 10–12	V39	L 17–28	V71	L 28–29	V103	L 16–38
V8	L 14–16	V40	L 17–29	V72	L 29–30	V104	L 24–38
V9 *	L 17–18	V41	L 17–30	V73 *	L 01–31	V105	L 24–37
V10	L 19–25	V42	L 18–27	V74	L 31–32	V106	L 18–40
V11	L 22–24	V43	L 18–28	V75	L 32–33	V107	L 13–24
V12	L 20–22	V44	L 18–29	V76	L 13–33	V108	L 09–24
V13 *	L 21–23	V45	L 18–30	V77	L 02–34	V109	L 05–24
V14	L 01–22	V46	L 05–25	V78	L 16–34	V110	L 01–24
V15	L 05–22	V47	L 09–25	V79	L 03–35	V111	L 01–13
V16	L 09–22	V48	L 13–25	V80	L 15–35	V112	L 36–37
V17	L 13–22	V49	L 01–19	V81	ANGLE AT INTER 01&05–09&13	V113	L 02–16
V18	L 22–31	V50	L 05–19	V82	ANGLE AT INTER 03&07–11&15	V114	L 03–15
V19	L 05–31	V51	L 09–19	V83	ANGLE AT INTER 05&01–24&20	V115	L 03–24
V20	L 22–33	V52 *	L 01–02	V84	ANGLE AT INTER 09&13–20&24	V116	L 07–24
V21	L 09–33	V53	L 02–03	V85 *	ANGLE AT INTER 03&01–01&13	V117	L 11–24
V22	L 22–32	V54	L 03–04	V86	ANGLE AT INTER 07&05–01&13	V118	L 15–24
V23 *	L 32–34	V55	L 01–04	V87	ANGLE AT INTER 11&09–01&13	V119	Area 1
V24	L 34–35	V56	L 05–06	V88 *	ANGLE AT INTER 15&13–13&01	V120	Area 2 *
V25	L 26–35	V57	L 06–07	V89 *	ANGLE 01-22-05	V121	Area 3 *
V26	L 01–05	V58	L 07–08	V90	ANGLE 05-22-09	V122	Area 4 *
V27	L 05–09	V59 *	L 05–08	V91	ANGLE 09-22-13	V123	Area 5
V28	L 09–13	V60	L 09–10	V92	ANGLE O2-25-19	V124	Area 6
V29	L 13–19	V61	L 10–11	V93 *	ANGLE 16-19-25	V125	Area 7
V30 *	L 19–20	V62	L 11–12	V94	ANGLE 01-24-05	V126	Area 8
V31	L 24–25	V63	L 09–12	V95 *	ANGLE 05-24-09	V127	Area 9
V32	L 01–25	V64	L 13–14	V96	ANGLE 09-24-13	V128	Area 10

**Fig 5:**
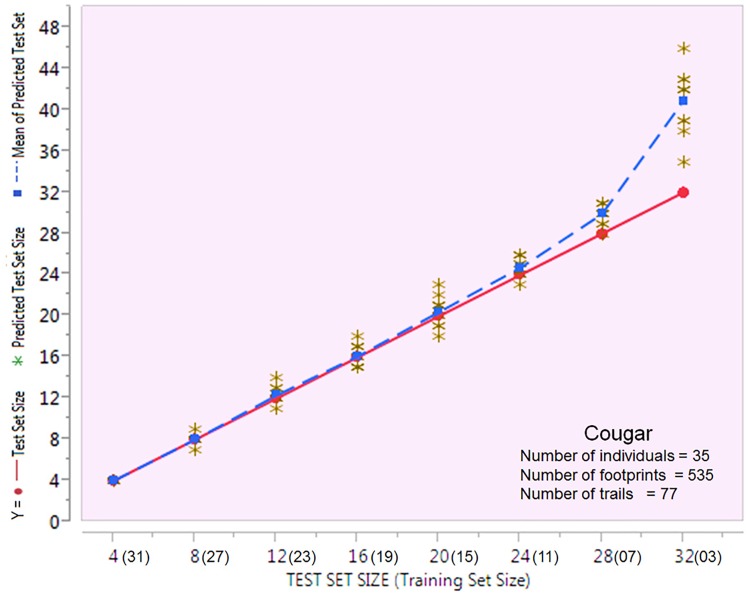
The figure shows a model validation of the accuracy of classification in relation to the size of the test set against the training set. The varying test set size was plotted against itself (red), the predicted value for each test size iteration (black) and the mean predicted value for each test size (blue). Optimal classification accuracy was obtained when the test set size was smallest relative to the training set. However, the robustness of the model was demonstrated by the predicted value being close to the expected value, even when the test set was considerably larger than the training set (24:11).

The numbers 01–25 refer to the landmark points and 26–40 to the derived points (see Fig 5).

Variables 81–88 were generated at the intersection of two vectors e.g. V81 refers to vector from points 01 to 05 and 09 to 13.

The areas were generated using the most peripheral points in each case. Area 1 = whole image; Area 2 = toe 5; Area 3 = toe 4; Area 4 = toe 3; Area 5 = toe 2; Area 6 = pad; Area 7 = points 1, 5, 9, 13 and the proximal pad points; Area 8 = points 1, 13 and the proximal pad points; Area 9 = points 1, 5, 9, 13 and points 19 & 25; Area 10 = points 1 and 13 to the most distal toe points (3, 7, 11, 15).

* = 20 variables selected stepwise for discriminating sex.

A pairwise robust cross-validated discriminant analysis is then performed. From the main FIT window, the ‘robust cross-validated pairwise analysis’ window is selected. The model scripted into the analysis uses a classifier to determine the likelihood of a pair of trails belonging to the same individual or two different individuals.

We then conduct a pairwise comparison of trails. This is detailed in Jewell et al. [28,62], using the training database of known individuals, and summarized below.

The FIT system produces two outputs, an assigned self/non-self table to describe the classification distance between each validation pair, and a classification matrices window showing the different trails selected for comparison. The software calculates the distances between any two trails, and produces a cluster dendrogram to classify them. The user can test the accuracy of classification by adjusting the number of variables and the contour probability. Adjusting the threshold value identifies the algorithm that consistently gives the highest accuracy to be selected.

A full holdback trial for validation is then performed To validate the algorithm, we divide the dataset into training and test sets, then conduct comprehensive sequential ‘holdback’ trials. Holdback is a validation method that randomly divides the original data into ‘training’ and ‘test’ data sets so that different compositions and sizes of subsets of ‘test’ trails can be held back as ‘unknown’ and classified against the remainder of the dataset (the training set).

Individual puma in the dataset are apportioned randomly to either the test or training sets. Holdback trials are then conducted to assess the accuracy of the algorithm for the expected number of individuals and the clustering classification.

### 2.6 FIT classification by sex

A standard discriminant analysis in FIT is used to classify footprints by the sex of the animal that made them.

We excluded the possibility of sex determination being linked to age by using a wide range of puma ages, and testing for age effects. For sex identification, we used 535 footprints from 35 captive pumas, 19 females (300 footprints) and 16 males (235 footprints). Animal ages ranged from 1 year to 20 years for both sexes without significant age differences between groups (Female mean age 9.13 yrs, Male mean age 12.63 yrs (Table 2).

**Table 2:** Footprint collection.

	# of individuals (known age)	Mean age (range)	# of footprint images	Mean # of footprints/individual (range)	# of trails	Mean trails/individual (range)
Females	19 (14)	9.13 (1–14.3)	300	15.78 (6–23)	45	2.37 (1–3)
Males	16 (13)	12.63 (1–20)	235	14.69 (7–25)	34	2.13 (1–4)
Total	35 (27)	10.81 (1–20)	535	15.29 (6–25)	79	2.26 (1–4)

The number of individual puma from whom footprint images were collected, their sex, their mean age (range), the total number of footprint images collected, the mean number of footprints per individual (range), the number of trails, and the mean number of trails per individual (range).

We used linear discriminant analysis in JMP to identify a linear combination of variables that characterize or separate different classes of objects, in this case sex. We used the JMP Stepwise Variable Selection function to select the variables that provided best discriminating power based on their F ratios. This function also has the added advantage of excluding highly correlated variables. By plotting the number of variables included in the analysis against the predicted level of accuracy of sex identification, we were able to establish the number of variables that would provide an effective predictive model.

To validate the level of accuracy of sex discrimination using linear discriminant analysis, we used 2 holdout methods, Jackknife and partitioning the data into training and test sets. Jackknifing tests sequential subsets of data, excluding every footprint in the data set in sequence, where partitioning splits the data to enable testing of one set against an algorithm derived from the other.

An algorithm derived from all the variables, consisting of the 10 best discriminating variables generated by the captive puma footprint data was used as a predictive model to determine sex for unknown free-ranging puma footprint data.

## 3 Results

### 3.1 Classification by individual

We collected 535 footprints from 35 individuals (19 females and 16 males). The number of puma individuals, the number of footprint images collected, the range of footprints per puma, the number of trails, the range of trails per puma and the mean and standard error of age per group are shown in Table 2.

The accuracy of individual identification was assessed using two different methods:

#### 3.1.1 Data validation model accuracy

Using the holdback method, we conducted 10 random iterations for each training/test subset size (Fig 5). The varying test set size was plotted against itself (red), the predicted value for each test size iteration (black) and the mean predicted value for each test size (blue). We demonstrate that even when the footprint test set size was increased considerably in relation to the training set size, the mean predicted value proved similar to the expected value.

Optimal accuracy in classifying individuals was obtained when the test set consisted of 4 animals relative to the training set of 31 animals, but even when the test set was 28 animals and the training set 7 animals, classification accuracy was 93.2%.

#### 3.1.2 Clustering accuracy

**Fig 6:**
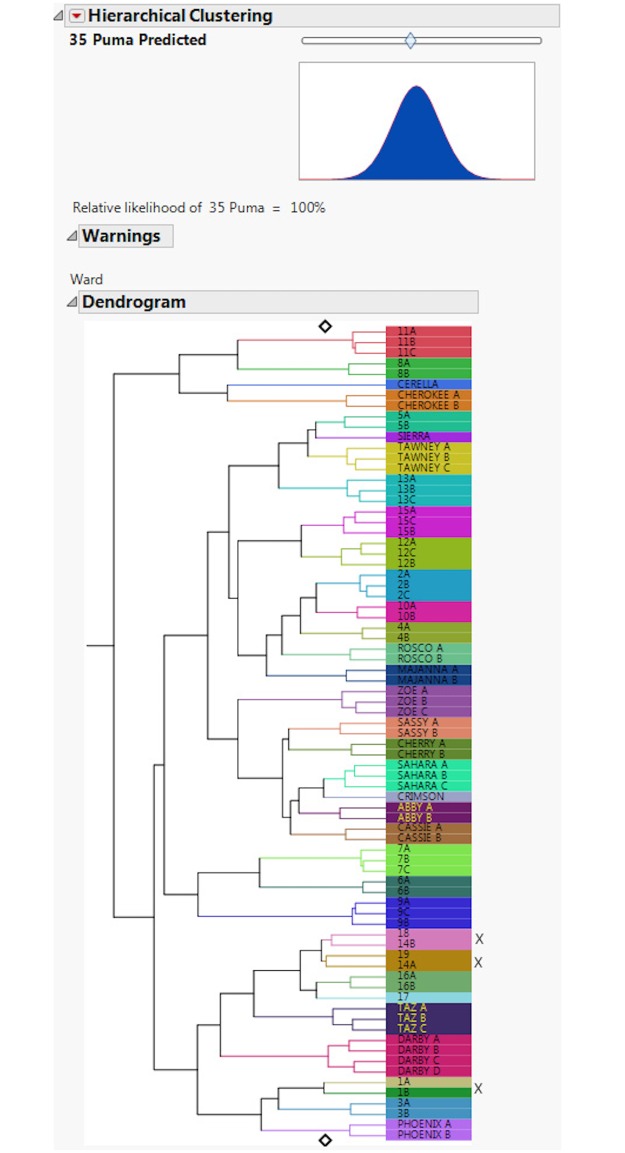
This figure shows the classification dendrogram output from FIT. Three trails of 77 were misclassified (indicated by an x), giving an overall accuracy of individual identification for correct trail placement of 96.11%.

**Fig 7:**
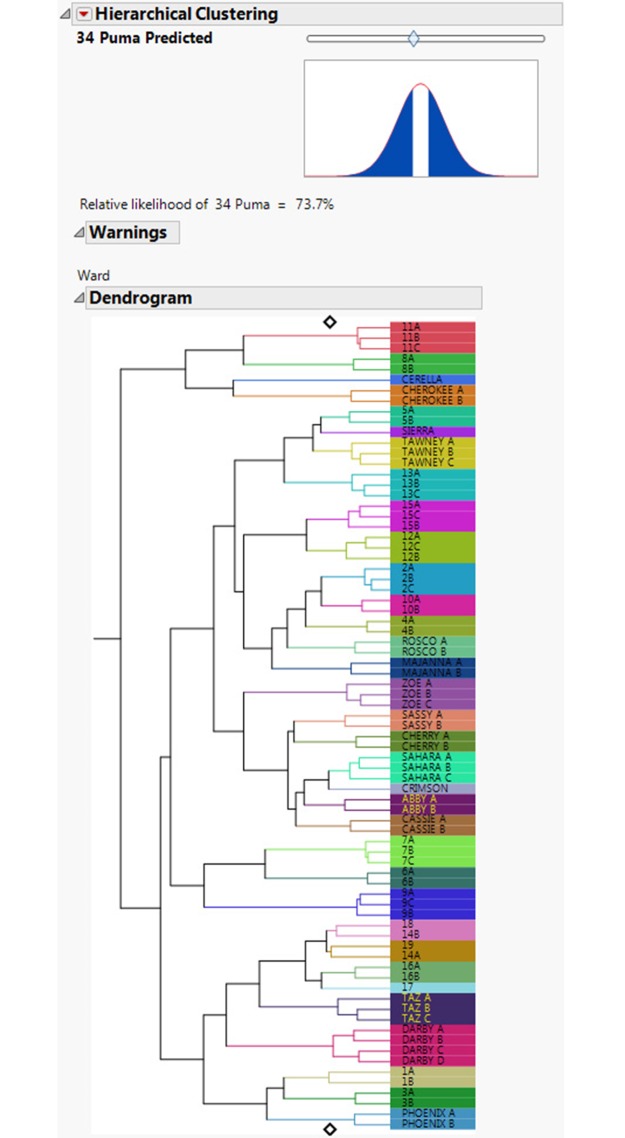
This figure shows the output when a relative likelihood slider is moved to the left, giving the relatively likelihood of 34 puma as 73.7%.

**Fig 8:**
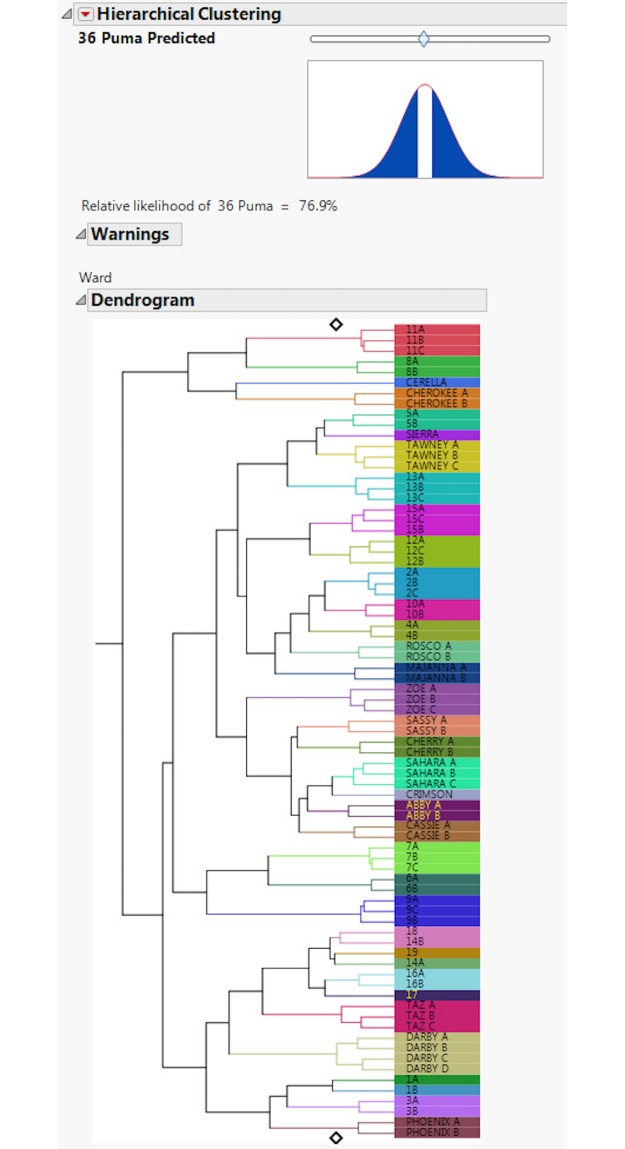
This figure demonstrates that when the slider is moved to the right the likelihood of 36 cougars is 76.9%.

**Fig 9:**
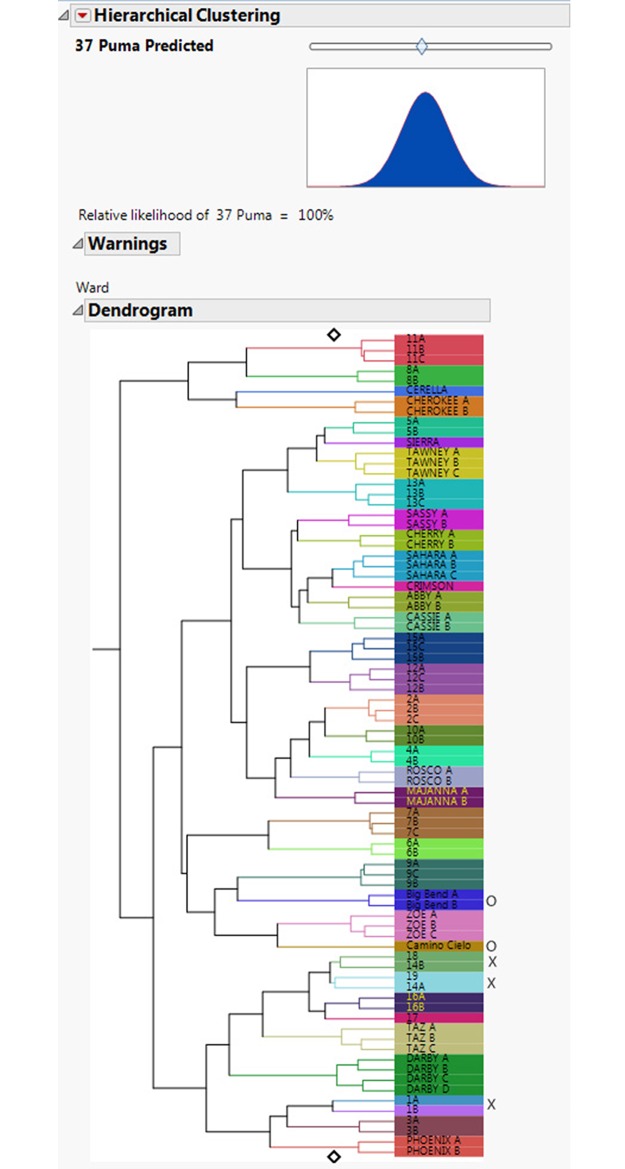
Footprints collected from free ranging animals at Big Bend and Camino Cielo (labeled o) are classified correctly as two individuals different from each other and from all the others in the dataset. Misclassified trails are again indicated by an x.

This is an assessment of how often individual footprint trails were assigned to the correct individual. A dendrogram (Fig 6) shows three trails out of 77 were misclassified, giving an overall individual accuracy for correct trail placement of 96.11%. The FIT model also allows for an assessment of relative likelihood of estimated puma numbers using a slider (Figs 7 & 8). The slider position is determined from a hypothesis test where the null hypothesis is the number of individuals set by the slider. Footprints collected from two additional free-ranging animals were then included in the test sets, and accurately separated out as two individuals (Fig 9).

### 3.2 Classification by sex

**Fig 10:**
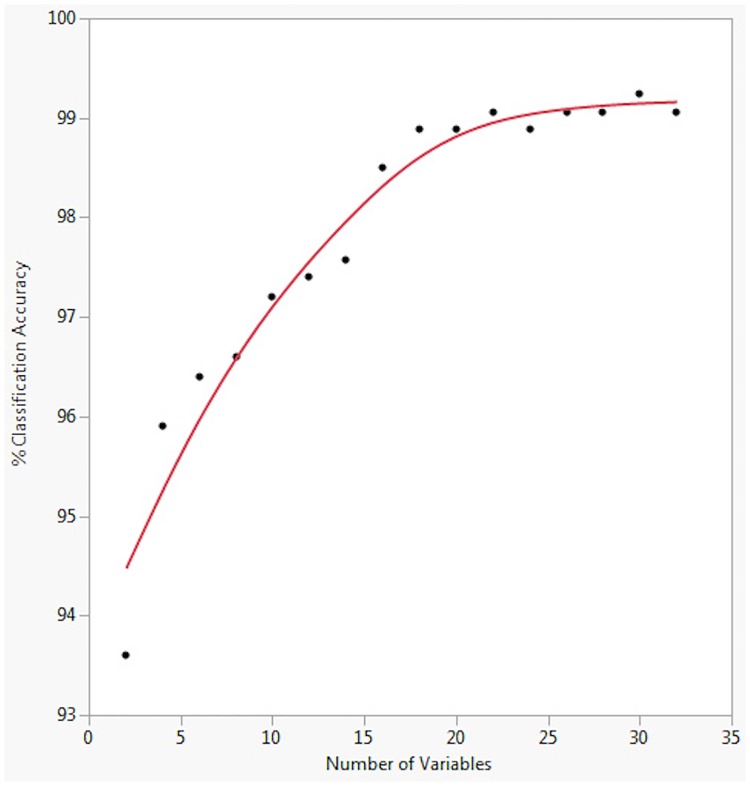
The relationship between the number of variables used to classify by sex and the resulting percentage accuracy of classification, from which 20 was identified as an optimal number.

The variables with the highest discriminating power were selected using the stepwise selection feature in JMP. To avoid using too many variables and over-fitting we used linear discriminant analysis to plot the number of variables against the sex-prediction for all the footprints. The optimal number of variables for individual classification was 20 (Fig 10).

**Fig 11:**
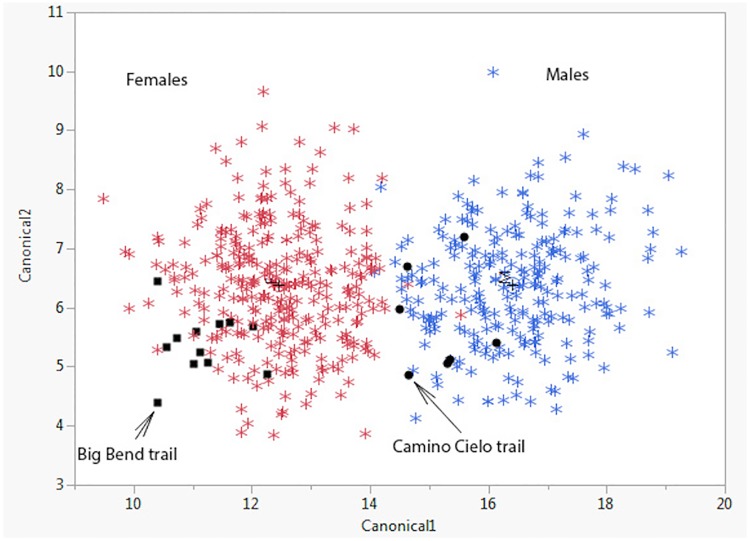
Scatterplot showing the distribution of footprints by sex. Red stars are footprints from females, and blue from males. The black squares and circles are from animals of unknown sex from Big Bend (classified as female) and Camino Cielo (classified as male) respectively.

The classification by sex was 99% accurate from a single footprint. In addition, two free-ranging animals were classified; the first as a male (from Camino Cielo, Los Padres National Forest in California) and the second as a female (from Big Bend National Park in Texas) using the algorithm developed from the captive animals (Fig 11).

## 4. Discussion

### 4.1 Potential applications

The FIT is an adaptable non-invasive tool. Footprints can be collected systematically in a small closed population to obtain an absolute count, or opportunistically throughout a larger or unfenced range area to obtain a minimum known alive (MKA) population estimate. Footprints can also be used as a ‘mark’ in a capture-recapture (CR) population estimate. Work is underway to validate the FIT for puma in snow substrates, as has already been achieved for Amur tiger [56], giant panda and Polar bear (Jewell, pers.comm.).

Footprints are generally the most ubiquitous animal sign, and data collection is simple, inexpensive and accessible to a range of data collecting groups from expert trackers and professional conservation biologists, to citizen scientists. The FIT can also be used synergistically with camera-traps. While trail cameras are of limited value for identifying individual pumas [66], the FIT could be combined with trail cameras to improve confidence. For example, the substrate on the ground covered by a camera trap can be prepared to allow footprints to be collected as the animal walks through. If traps are checked frequently (at least every 24 hours) then whole animal images could be matched to footprints.

### 4.2 Potential limitations

This methodology requires good quality footprints, which require suitable impression-holding substrate. In some environments it may not be possible to find enough high quality footprints for this methodology to be easily applied. However, it may still be possible to create “footprint traps” by placing sand in travel corridors or by raking and smoothing the existing substrate.

While limited tracking skills are needed to collect photos for the FIT, it is still necessary for trackers to be able to accurately identify puma left hind footprints. Even experienced biologists can struggle with footprint identification [27]. However, in our experience, most observers were able accurately identify left hind puma footprints after a day of training. The benefit of using digital images to collect the footprints is that they can be independently verified for accuracy at a later time by a skilled observer.

### 4.3 Future development

This paper has demonstrated the development of the FIT puma algorithm from captive puma, and a potential application to monitor free-ranging puma and other large felids. The next step is to undertake wider field validation of free-ranging puma on various substrates.

## Supporting information

S1 Table WildTrack mountain lion footprint dataset (electronic file). The full set of measurements taken from each footprint of each individual.
(XLSX)

S1 Video Spotting cheetahs: Identifying individuals by their footprints. A step-by-step guide to using the Footprint Identification Technique.
(MP4)

## Abbreviations

F.I.T.: Footprint Identification Technique
