# Correction: The challenge of monitoring elusive large carnivores: An accurate and cost-effective tool to identify and sex pumas (*Puma concolor*) from footprints

**DOI:** 10.1371/journal.pone.0242584

**Published:** 2020-11-12

**Authors:** Sky Alibhai, Zoe Jewell, Jonah Evans

After this article [1] was published, concerns were raised that the software described in the article had not been made publicly available.

The *PLOS ONE* article [1] reports an application of a Footprint Identification Technique (FIT) algorithm for puma, but there is no specific puma software instantiation of FIT. The FIT software has been reported previously in the context of its development for monitoring other species [2–4], and is an add-in to commercial software JMP (JMP.com). This article [1] uses the same FIT code as for other species but incorporates variables to identify puma; the puma-specific variables are reported in Table 1 of [1]. Since the code was not newly developed for the *PLOS ONE* study it is not subject to the terms of the journal’s Software Sharing Policy.

The FIT process, including the underlying scripts, has been patented [5] and is not publicly available. However, the authors are happy to collaborate with interested researchers and will provide FIT free of charge provided those requesting the software receive free training from the authors on how to use it.

The article’s Competing Interests statement is updated to:

Authors SA and ZJ hold a patent for the Footprint Identification Technique (FIT), including the underlying algorithms and scripts (U.S. Patent No. 9,911,066, March 6, 2018).
